# Clinical, dietary and demographic characteristics interfering on quality of life of cancer patients

**DOI:** 10.31744/einstein_journal/2018AO4368

**Published:** 2018-11-29

**Authors:** Juliana Alvares Duarte Bonini Campos, Wanderson Roberto da Silva, Maria Claudia Bernardes Spexoto, Sergio Vicente Serrano, João Marôco

**Affiliations:** 1 Universidade Estadual Paulista “Júlio de Mesquita Filho”, Araraquara, SP, Brazil.; 2 Hospital de Câncer de Barretos, Barretos, SP, Brazil.; 3 Instituto Universitário de Ciências Psicológicas, Sociais e da Vida, Lisboa, Portugal.

**Keywords:** Dietetics, Eating, Neoplasms, Quality of life, Dietética, Ingestão de alimentos, Neoplasias, Qualidade de vida

## Abstract

**Objective:**

To estimate the dietary intake of cancer patients and its relation with clinical and demographic characteristics, and to assess the contribution of dietary intake, appetite/symptoms and clinical and demographic characteristics to their quality of life.

**Methods:**

The consumption of energy and macronutrients of patients was estimated. The relation between dietary intake and clinical and demographic characteristics was evaluated by analysis of variance. The intake of energy and macronutrient of the patients was compared to the nutritional recommendations using 95% confidence interval. The Cancer Appetite and Symptom Questionnaire (CASQ) and the European Organization for Research and Treatment of Cancer (EORTC QLQ C-30) were used to assess appetite/symptoms and quality of life, respectively. The psychometric properties of the instruments were estimated. A structural equation model was prepared.

**Results:**

In this study, 772 cancer patients (63.1% women) participated. There was a significant relation between dietary intake and work activity, economic class, specialty field of cancer, type of treatment and nutritional status. Patients’ energy and macronutrients intake was below recommended values. Both CASQ and EORTC QLQ C-30 were refined to fit the data. In the structural model, impaired appetite, more symptoms, presence of metastasis, being female and of higher economic classes were characteristics that significantly contributed to interfering in patients’ quality of life.

**Conclusion:**

The dietary intake of oncology patients did not reach the recommended values. Different characteristics impacted on quality of life of patients and should be considered in clinical and epidemiological protocols.

## RESUMO

**Objetivo:**

Estimar a ingestão dietética de pacientes com câncer e sua relação com características clínicas e demográficas, além de verificar a contribuição da ingestão dietética, apetite/sintomas e características clínicas e demográficas para a qualidade de vida deles.

**Métodos:**

Foi estimado o consumo de energia e de macronutrientes. A relação entre ingestão dietética e características clínicas e demográficas foi avaliada pela análise de variância. A ingestão de energia e macronutrientes dos pacientes foi comparada com a necessidade nutricional, utilizando intervalo de confiança de 95%. O *Cancer Appetite and Symptom Questionnaire* (CASQ) e o *European Organization for Research and Treatment of Cancer* (EORTC QLQ C-30) foram utilizados para avaliar apetite/sintomas e qualidade de vida, respectivamente. As propriedades psicométricas dos instrumentos foram estimadas. Elaborou-se modelo de equações estruturais.

**Resultados:**

Participaram do estudo 772 pacientes oncológicos (63,1% mulheres). Observou-se relação significativa entre ingestão dietética e atividade laboral, classe econômica, especialidade do diagnóstico, tipo de tratamento e estado nutricional. O consumo de energia e macronutrientes dos pacientes esteve abaixo do recomendado. Tanto o CASQ quando o EORTC QLQ C-30 foram refinados para bom ajustamento aos dados. No modelo estrutural, comprometimento do apetite, maior acometimento por sintomas, presença de metástase, ser mulher e pertencer às classes econômicas mais altas foram características que contribuíram significativamente para o comprometimento da qualidade de vida dos pacientes oncológicos.

**Conclusão:**

A ingestão dietética dos pacientes oncológicos não atingiu os níveis recomendados. Diferentes características impactaram na qualidade de vida dos pacientes e devem ser consideradas em protocolos clínicos e epidemiológicos.

## INTRODUCTION

The term “quality of life” is often investigated and discussed by different researchers/professionals from different areas. In an attempt to unify this term, the World Health Organization (WHO) defined quality of life as “an individual’s perception of their position in life in the context of the culture and value systems in which they live and in relation to their goals, expectations, standards and concerns”.^(^ ^1^ ^)^ Therefore, quality of life can be considered a complex and multidimensional concept sensitive to physical, psychological, social and environmental changes.^(^ ^2^ ^)^ There is an increasing interest by researchers in improving the health-related quality of life of individuals, especially those affected by diseases.

Cancer is a disease frequently investigated in the context of quality of life. Its prevalence increases over the years, with a negative impact in the lives of people. Patients suffering from severe diseases, such as cancer, usually present with a variety of symptoms, such as nausea, vomiting, diarrhea, constipation, eating aversions, fatigue, dyspnea, and pain, which can influence their daily lives.^(^ ^2^ ^-^ ^4^ ^)^ It is likewise noted that appetite is also an aspect that can interfere in the daily life of these patients. Cancer patients undergoing oncologic treatment, mainly chemotherapy, report experiencing changes in both taste and appetite.^(^ ^5^ ^-^ ^9^ ^)^

Another important point to note is that cancer associated with appetite changes and the toxic effects of treatment may present increased severity and persistence of symptoms, affecting the patients’ dietary intake^(^ ^3^ ^,^ ^10^ ^-^ ^12^ ^)^ and, consequently, their quality of life.^(^ ^13^ ^,^ ^14^ ^)^

In addition, tumor site, the clinical stage of the disease, some symptoms, and the use of chemotherapy/radiation therapy are associated with changes in dietary intake.^(^ ^9^ ^,^ ^15^ ^)^ However, few studies reported results on the influence of dietary intake on the quality of life of cancer patients.

Some studies^(^ ^9^ ^,^ ^16^ ^-^ ^18^ ^)^ suggested that low food intake may influence the ability of cancer patients to maintain an adequate nutritional status during treatment, but few works included a quantitative analysis of energy and macronutrient intake, assessment of appetite and symptoms, and the relations between these aspects and quality of life of cancer patients. Although the influence of clinical and demographic characteristics on quality of life has been investigated and well documented in the literature, these relations have not been simultaneously correlated with energy/macronutrient intake and appetite, which renders the present investigation relevant.

## OBJECTIVE

To estimate energy and macronutrient intake and its relation with clinical and demographic characteristics in cancer patients; to compare energy and macronutrient intake with current recommendations for cancer patients; and to assess the influence of dietary intake, appetite, symptoms, treatment, and clinical and demographic characteristics on quality of life of cancer patients.

## METHODS

### Study design and sample size

This was a cross-sectional study with a non-probabilistic sampling design for convenience. The minimum sample size was calculated so as to ensure at least five individuals per parameter, in the hypothesized model.^(^ ^19^ ^)^ The final model tested consisted of 76 parameters, which resulted in an initial estimate of 380 individuals. However, we added a loss rate of 20%, raising the minimum sample size to 456 individuals.

### Participants

An invitation to participate in the study was extended to patients diagnosed with malignant neoplasm treated at the *Hospital de Câncer de Barretos*, in Barretos (SP). The exclusion criteria were patients who had undergone surgical procedures of high and intermediate complexity, individuals with cognitive impairment, and patients aged under 18 years. Only those who signed the informed consent were included in the study sample.

### Study variables

The demographic data collected included sex, age, marital status, religious beliefs and practice, work activity, and socio-economic class. Age was assessed in full years. Marital status was categorized into single, married, widow/er, and separated/divorced. Religious practice and working activity were evaluated dichotomically (yes or no). The socio-economic class was obtained using the Brazilian Economic Classification Criteria.^(^ ^20^ ^)^

Clinical information on the disease was obtained from the patient’s medical record. The variables evaluated were type of neoplasm (specialty field of cancer), stage of disease, type of treatment (chemotherapy, radiation therapy, or chemotherapy and radiation therapy), and metastasis (yes or no).

Weight and height reported by the patient were recorded for calculating body mass index (BMI) and for a subsequent classification of the anthropometric nutritional status.^(^ ^21^ ^)^

Dietary intake, appetite/symptoms and quality of life were estimated using the specific tools described below.

### Measuring instruments

#### Food Frequency Questionnaire

Dietary intake was estimated using the Food Frequency Questionnaire (FFQ) proposed by Matarazzo et al.,^(^ ^22^ ^)^ for cancer patients. Participants were asked to report the daily frequency of all food and drink intakes.

Energy, protein, lipid, and carbohydrate intake was estimated using the Brazilian Food Composition Table (TACO). For food items whose composition was not described in TACO, the AVANUTRI 4.0 program and the Brazilian Food Composition Table were used.

#### Cancer Appetite and Symptom Questionnaire

Patients’ appetite changes and symptoms were evaluated using the Cancer Appetite and Symptom Questionnaire (CASQ), originally proposed by Halliday et al.,^(^ ^23^ ^)^ This questionnaire comprised 12 items (4 reverse scored), using a 5-point Likert scale in a unifactorial model. A Portuguese version of CASQ was developed and presented by Spexoto et al.,^(^ ^24^ ^)^ who reported appropriate psychometric indicators when the tool was applied to cancer patients.

#### European Organization for Research and Treatment of Cancer − Quality of Life Questionnaire Core 30

Quality of life was assessed using the European Organization for Research and Treatment of Cancer - Quality of Life Questionnaire Core 30 (EORTC QLQ-C30), originally proposed by Aaronson et al.,^(^ ^25^ ^)^ The tool comprised 30 items, using a 4-point and 7-point Likert scale and 10 factors.

The factors were General Quality of Life (items: 19 and 30), Physical Function (items: 1, 2, 3, 4, and 5), Functional Performance (items: 6 and 7), Emotional Function (items: 21, 22, 23, and 24), Cognitive Function (items: 20 and 25), Social Function (items: 26 and 27), Fatigue (items: 10, 12 and 18), Nausea/Vomiting (items: 14 and 15), Pain (items: 9 and 19), and Spurious Conditions (items: 8, 11, 13, 16, 17, and 28).

A Portuguese version of EORTC QLQ-C30 was developed and presented by the European Organisation for Research and Treatment of Cancer. Campos et al.,^(^ ^26^ ^)^ reported adequate psychometric indicators using the Portuguese version of the tool on cancer patients.

## Statistical analysis

An analysis of variance (ANOVA) was used to compare dietary intakes, according to clinical and demographic characteristics. The dependent variable was dietary intake (represented by daily energy, protein, lipid, and carbohydrate intake), and the independent variables were clinical and demographic characteristics. The Tukey’s post-test was used for multiple comparisons and, when homoscedasticity was violated, the Welch’s correction was used, followed by the Games-Howell post-test.

To compare the patients’ energy, protein, lipid, and carbohydrate intake with reference values, a 95% confidence interval was used (95%CI). The patients’ energy and protein intake was compared to the recommendations of the National Consensus on Cancer Nutrition of the *Instituto Nacional de Câncer José Alencar Gomes da Silva* (INCA),^(^ ^27^ ^)^ whereas lipid and carbohydrate intake was compared to the recommendations of the Institute of Medicine (IOM).^(^ ^28^ ^)^

Cancer Appetite and Symptom Questionnaire and EORTC QLQ-C30 psychometric properties were estimated for the study sample. A confirmatory factor analysis was performed for the estimation using the Weighted Least Squares Mean and Variance Adjusted (WLSMV) estimator in a polychoric correlation matrix. χ^2^ ratio indexes to degrees of freedom (χ^2^/df), root mean square error of approximation (RMSEA), comparative fit index (CFI), and Tucker-Lewis Index (TLI) were used to assess the tools’ adjustment. Values of χ^2^/df≤5.0, RMSEA≤0.10, CFI and TLI≥0.90 were considered adequate.^(^ ^29^ ^)^ When the tools did not present adequate fit to the sample, we used the modification indexes estimated using Langrange Multipliers (LM) for their refinement.^(^ ^29^ ^)^ The factorial weight (λ) of each tool item was also evaluated, and values >0.35 were considered acceptable.^(^ ^19^ ^)^ The internal consistency was also investigated using the Cronbach’s alpha coefficient, and values greater than 0.70 were considered adequate.

A hypothetical causal model was constructed, considering “quality of life impairment” as a dependent variable. For this, a second order hierarchical model of EORTC QLQ-C30 was prepared. The first-order factor “General Quality of Life” was not included in the analyses, because it deals with a generalized evaluation. The variables energy, protein, lipid, carbohydrate, BMI, stage of the disease (1=I, 2=II, 3=III, or 4=IV), metastasis (0=no,1=yes), sex (0=female, 1=male), religious practice (0=no, 1=yes), work activity (0=no, 1=yes), and socio-economic class (1=D and E, 2=C, 3=B, 4=A) were included in the model as independent variables.

The model was prepared using the software MPLUS 7.2 (Muthén & Muthén, Los Angeles, 2014) and the WLSMV estimator. The measurement model was adjusted using χ^2^/df, RMSEA, CFI, and TLI indexes, with their respective reference values. The significance of the hypothetically causal trajectories (β) was also evaluated (z-test).^(^ ^29^ ^)^ The significance level was set at 5% for decision making. To refine the model, we considered only the significant trajectories evaluated step by step. In addition, a multicollinearity investigation was performed by calculating the variance inflation factor (VIF), and values > 5 were considered as indicative of multicollinearity.

## Procedures and ethical aspects

Properly trained researchers collected data in the waiting rooms and inpatient units of the *Hospital de Câncer de Barretos*. The data collection period was from 2013 to 2014, and patients were interviewed while waiting for care. Participation was voluntary, and patients received all information regarding the study objectives and ethical aspects, and anonymity was guaranteed. The patients’ clinical information was collected from their medical records.

This study was approved by the Himan research Ethics Committee of the *Hospital de Câncer de Barretos* (protocol 561/2011).

## RESULTS

A total of 772 cancer patients of both sexes (63% women) were included in the study. The mean age of participants was 53.2 (standard deviation of 12.7) years, and the mean BMI was 25.8kg/m^2^ (standard deviation of 5.4). Table 1 shows the patients’ clinical and demographic characteristics, comparing dietary intakes, according to these characteristics.

**Table 1:** Demographic and clinical characteristics of the sample of cancer patients and comparison of dietary intake

Characteristic	Dietary intake (mean±standard deviation)								

	n (%)	Total energy (kcal/day)	p value	Protein (g/day)	p value	Lipid (g/day)	p value	Carbohydrate (g/day)	p value
Sex									
Male	286 (37.0)	1,330.70±347.49		57.38±22.05		27.25±10.23		218.46±55.35	
Female	486 (63.0)	1,355.82±322.56	0.311	56.79±18.62	0.691	27.39±9.75	0.844	226.19±55.70	0.063
Religion									
No	29 (3.8)	1,289.67±372.71		56.72±21.30		26.13±8.84		211.27±58.61	
Yes	743 (96.2)	1,348.76±330.37	0.347	57.02±19.91	0.937	27.39±9.97	0.504	223.81±55.53	0.234
Religious practice									
No	107 (13.9)	1,289.88±343.95		55.17±20.66		25.67±9.40		214.38±57.44	
Yes	665 (86.1)	1,355.66±329.37	0.057	57.30±19.83	0.305	27.61±10.00	0.061	224.78±55.28	0.073
Marital status									
Single	112 (14.5)	1,340.85±333.35		58.80±20.67		28.20±10.73		217.67±55.03	
Married	506 (65.6)	1,357.95±335.58		57.25±20.05		27.29±9.88		226.12±55.95	
Widow/er	77 (10.0)	1,350.25±344.61		56.64±19.61		28.23±10.12		223.06±59.82	
Separated/divorced	76 (9.9)	1,275.89±289.59	0.253	53.17±18.50	0.284	25.49±8.70	0.251	213.53±49.67	0.188
Work activity									
No	558 (72.3)	1,327,19±322.63		55.46±19.31		26.52±9.24		221.83±54.26	
Yes	214 (27.7)	1,397.02±350.98	0.009*	61.04±21.04	<0.001*	29.48±11.27	<0.001*	227.27±59.12	0.224
Economic class, mean income									
A (R$ 9,263.00)	20 (2.6)	1,435.64±345.32^a^		66.96±25.64^a^		32.33±12.64^a^		224.85±53.66^a.b^	
B (R$ 3,947.00)	267 (34.6)	1,384.53±306.26^a^		59.67±19.34^a^		28.15±9.43^a^		228.86±52.03^a^	
C (R$ 1,416.00)	368 (47.7)	1,349.66±343.88^a^		56.49±19.90^a^		27.32±10.23^a.b^		224.50±57.14^a^	
D and E (R$ 776,00)	117 (15.1)	1,234.84±326.26^b^	<0.001*	50.88±18.81^b^	<0.001*	24.71±9.00^b^	0.002*	206.81±56.86^b^	0.004*
Specialty field of cancer									
Head and neck	61 (7.9)	1,400.15±386.66		60.45±24.85^a,b^		29.34±12.20^a,b^		228.00±60.00	
Upper digestive tract	81 (10.5)	1,425.29±397.32		62.59±25.56^a^		29.51±11.33^a^		232.55±65.77	
Lower digestive tract	175 (22.7)	1,363.60±304.37		58.45±19.62^a,b^		28.27±8.87^a^		223.42±50.66	
Gynecology	102 (13.2)	1,367.54±359.64		57.80±19.92^a,b^		28.30±10.97^a,b^		225.97±62.10	
Breast cancer	248 (32.1)	1,314.95±281.99		54.34±16.23^a,b^		25.95±8.98^a,b^		221.85±49.31	
Lung	46 (6.0)	1,250.66±360.45		50.11±18.56^b^		24.10±7.91^b^		213.60±62.37	
Urology	59 (7.6)	1,303.70±351.09	0.065^†^	56.73±19.54^a,b^	0.008^†^	26.24±10.39^a,b^	0.003^†^	214.87±58.27	0.592^†^
Stage of disease									
I	51 (6.6)	1,296.30±268.54		54.82±14.33		26.34±8.29		215.31±44.09	
II	196 (25.4)	1,321.71±318.07		55.92±18.39		26.48±9.52		219.89±56.03	
III	301 (39.0)	1,356.92±331.54		57.50±20.03		27.72±10.16		224.60±55.58	
IV	224 (29.0)	1,365.77±356.22	0.347	57.79±22.16	0.632	27.80±10.28	0.407	226.47±57.77	0.445
Type of treatment									
Chemotherapy	559 (72.4)	1,351.59±336.11^a^		57.28±20.26^a^		27.45±10.01^a^		224.20±56.52	
Radiation therapy	95(12.3)	1,265.77±282.07^a^		50.39±13.78^b^		23.95±7.20^b^		217.76±50.90	
Chemotherapy and radiation therapy	118 (15.3)	1,387.66±341.22^b^	0.023*	61.06±21.42^a^	<0.001*	29.53±10.76^a^	<0.001*	223.71±55.41	0.579
Presence of metastasis									
No	459 (59.5)	1,340.53±319.58		56.63±19.22		27.29±9.89		222.15±52.57	
Yes	313 (40.5)	1,355.37±349.69	0.542	57.56±20.99	0.529	27.41±9.99	0.877	225.07±59.96	0.476
Body mass index									
<18.5 (low weight)	52 (6.8)	1,415.38±416.95		61.69±26.09		30.74±11.14^a^		228.28±63.51	
18.5|-25.0 (eutrophy)	326 (42.2)	1,368.17±349.23		58.21±20.81		28.26±10.36^a^		225.55±58.86	
25.0|-30.0 (overweight)	244 (31.6)	1,307.09±295.46		54.34±16.66		25.82±9.19^b^		219.40±51.74	
≥30.0 (obesity)	150 (19.4)	1,339.86±311.62	0.082^†^	57.10±20.16	0.050^†^	26.71 ±9.27^a,b^	0.004^†^	223.21±51.80	0.545^†^

Equal letters indicate statistical similarity. * Statistically significant difference (p<0.05); ^†^ analysis of variance with Welch correction (Games-Howell *post hoc* test).

There was a higher prevalence of female individuals, with religious beliefs and practice, married, with work activity, in socio-economic class C, and with eutrophic nutritional status. In addition, individuals with stage III breast cancer, undergoing chemotherapy, with no metastasis were prevalent. Dietary intake had a significant relation with work activity, socio-economic class, specialty field of cancer, type of treatment, and BMI. Individuals who reported having work activity had higher energy, protein, and lipid intake, and the opposite was true for individuals in the lower socio-economic classes. As to specialty field of cancer, patients with lung cancer had lower protein and lipid intake than patients with upper and lower gastrointestinal cancer. Patients undergoing chemotherapy and radiation therapy had greater energy intake than patients receiving other treatments. However, patients undergoing radiation therapy alone had lower protein and lipid intake. As to BMI, individuals classified as overweight presented lower lipid intake than those classified as eutrophic or underweight.

Table 2 shows a comparison between patients’ dietary intake and reference values for energy and macronutrient intake. The participants’ intakes were below recommended levels.

**Table 2:** Characterization of energy and macronutrient intake of cancer patients and recommended intake values

	Mean±standard deviation	Median	Mode	Asymmetry	Kurtosis	95%CI
Energy, kcal/day						
Intake	1,346.55±331.98	1.317.70	903.62	0.62	1.37	1,323.13-1,369.97
Recommendation*	1,716.22±391.58	1,675.00	1,700.00	0.68	0.68	1,688.60-1,743.84
Protein, g/day						
Intake	57.01±19.95	54.07	54.07	1.24	2.66	55.60-58.42
Recommendation*	82.38±18.80	80.40	81.60	0.68	0.68	81.05-83.71
Lipid, g/day						
Intake	27.34±9.92	25.86	21.02	1.04	2.06	26.64-28.04
Recommendation^†^	57.21±13.05	55.83	56.67	0.68	0.68	56.29-58.13
Carbohydrate, g/day						
Intake	223.33±55.66	222.87	206.50	0.46	1.19	219.40-227.26
Recommendation^†^	235.98±53.84	230.31	233.75	0.68	0.68	232.18-239.78

* Recommendation values, according to the National Consensus on Cancer Nutrition of the *Instituto Nacional de Câncer José Alencar Gomes da Silva*;^(27)†^ acceptable values of macronutrient distribution, as per the Institute of Medicine. 95%CI: 95% confidence interval.

Table 3 shows indicators for the assessment of psychometric properties, using CASQ and EORTC QLQ-C30. None of the tools presented appropriate fit to the data. After assessing the original proposals, the data were then refined. For CASQ, two items were excluded due to low factorial weights (item 5: λ=0.27; and item 6: λ=0.19). Three correlations were also added between item errors (1-2: LM=49.93; 1-3: LM=168.78; 10-11: LM=106.96).

**Table 3:** Indicators for assessment of psychometric properties of the Cancer Appetite and Symptom Questionnaire and the European Organization for Research and Treatment of Cancer - Quality of Life Questionnaire Core 30 applied to a sample of cancer patients

Instrument	Model	χ^2^/df	RMSEA (90CI%)	CFI	TLI	λ	β	EI	e	α
Original CASQ	Unifactorial	12.79	0.12 (0.11-0.13)	0.93	0.92	0.19-0.92	-	-	-	0.80
Refined CASQ	Unifactorial	7.72	0.09 (0.08-0.10)	0.98	0.97	0.36-0.92	-	5 e 6	1 and 2,	0.81
									1 and 3,	
									10 and 11	
Original EORTC QLQ-30	9 first-order factors	2.71	0.05 (0.04-0.05)	0.97	0.96	0.52-0.99	-	-	-	0.41-0.83
Refined EORTC QLQ-30	8 first-order factors	3.63	0.06 (0.05-0.06)	0.96	0.95	0.50-0.98	-	8, 11, 13, 16, 17, 28, 29 and 30	-	0.50-0.83
Refined EORTC QLQ-30	8 first-order factors and 1 second-order factor	4.11	0.06 (0.06-0.07)	0.95	0.94	0.50-0.98	0,53-0,94	8, 11, 13, 16, 17, 28, 29 and 30	-	0.50-0.83

χ^2^/gl: degree-of-freedom χ^2^ test; RMSEA: root mean square error of approximation; 90%CI: 90% confidence interval; CFI: comparative fit index; TLI: Tucker-Lewis index; λ: factorial weight of items; β: standardized estimates of trajectories; IE: excluded items; e: items with correlation among errors; α: Cronbach´s alpha coefficient;CASQ: Cancer Appetite and Symptom Questionnaire; EORTC QLQ-C30: European Organization for Research and Treatment of Cancer - Quality of Life Questionnaire Core 30.

For fit of EORTC QLQ-C30 to the sample, the Spurious factor was excluded. Then the hierarchical model was tested with a second order factor called “Quality of life impairment”. This model presented appropriate fit to the data. Internal consistency was adequate in both tools, with the exception of the EORTC QLQ-C30 “Cognitive function” factor.

Table 4 shows the structural model (complete and refined) tested with the hypothetically causal trajectories for the cancer patients sample. CASQ item 12 and the EORTC QLQ C-30 Pain factor were collinear (VIF=5.26), and this item was eliminated.

**Table 4:** Structural (complete and refined) model considering the impact of clinical and demographic variables, dietary intake and appetite/symptoms, on quality of life of cancer patients

Independent variable	Complete			Refined		
	
	β	EP	p value	β	SE	p value
Appetite/symptoms (CASQ)	0.766	0.022	<0.001*	0.645	0.027	<0.001*
Sex	-0.120	0.043	0.005*	-0.197	0.086	0.021*
Religious practice	-0.015	0.039	0.705	-	-	-
Work activity	-0.033	0.039	0.401	-	-	-
Stage of disease	0.062	0.048	0.192	-	-	-
Presence of metastases	0.174	0.046	<0.001*	0.423	0.078	<0.001*
Body mass index	-0.036	0.040	0.361	-	-	-
Economic class	0.087	0.040	0.028*	0.152	0.052	0.003*
Energy, kcal/day	0.637	1.123	0.570	-	-	-
Protein, g/day	-0.198	0.297	0.505	-	-	-
Lipid, g/day	-0.117	0.305	0.701	-	-	-
Carbohydrate, g/day	-0.503	0.717	-0.702	-	-	-

β: standardized estimate (trajectory); SE: standard error; CASQ: Cancer Appetite and Symptom Questionnaire. * p<0.05.

The complete model presented some non-significant trajectories and was refined. After refinement, the model presented adequate fit to the data (χ^2^/df=3.90; RMSEA=0.06; 90%CI 0.05-0.06; CFI= 0.92; TLI=0.91) and explained variance of 47%. It was observed that the greater the impairment of the appetite and the symptoms of the disease (CASQ), the greater the impairment of quality of life in cancer patients.

Metastasis, female sex and higher socio-economic classes were characteristics that contributed significantly to interfering in quality of life of the patients evaluated.

## DISCUSSION

Despite the importance the literature^(^ ^13^ ^,^ ^14^ ^)^ ascribes to food intake as an interfering factor in quality of life of cancer patients, the present study showed that this was not significant. On the other hand, there was a significant influence of appetite and symptoms of disease on patients’ quality of life. The same is true for clinical and demographic variables, which corroborates recent studies.^(^ ^4^ ^,^ ^30^ ^,^ ^31^ ^)^ These findings suggest that these factors overlap dietary intake and should be considered by healthcare professionals in intervention protocols, aiming at a more resolutive and focused treatment to improve the quality of life of cancer patients.

Although it had no impact on quality of life, an adequate dietary intake is important to maintain good health and improve the patient’s prognosis. The daily dietary intake of patients was below recommendations, which should be considered a concern.^(^ ^32^ ^)^ Fearon et al.,^(^ ^33^ ^)^ and Roxburgh et al.,^(^ ^34^ ^)^ attributed the cancer patients’ inadequate food intake to the tumor itself, whereas Jeffery et al.,^(^ ^18^ ^)^ and Pearce et al.,(^35^ ^)^ related this to the toxicity of the treatment. Other authors^(^ ^2^ ^,^ ^3^ ^,^ ^36^ ^,^ ^37^ ^)^ stated both tumor and treatment were able to impair the food intake. Energy intake of cancer patients may vary according to the type of disease, therapeutic protocol, prior nutritional status and complications; therefore each of these aspects should be evaluated individually.

The significant relations between dietary intake and clinical and demographic characteristics should also be discussed. The higher energy and macronutrient intake of individuals of higher socio-economic classes could be attributed to their higher purchasing power to buy food. On the other hand, the higher intake of energy, protein and lipid in individuals with some work activity could be associated to industrialized or out-of-home meals.^(^ ^38^ ^)^ It can also be speculated that working individuals may have a more favorable clinical condition with fewer symptoms and, hence, a better functional capacity, better appetite and less side effects of treatment, which can contribute to a better diet, resulting in higher intake of macronutrients and energy.

Additionally, individuals classified as overweight presented lower lipid intake than those classified as eutrophic or underweight. This result may be attributed to the fact that overweight individuals generally receive professional guidance based on the WHO proposals^(^ ^39^ ^)^ to limit energy intake from lipid.

Regarding the psychometric properties of the tools (CASQ and EORTC QLQ C-30), both had an appropriate fit for the study sample only after some modifications were made. Some studies^(^ ^24^ ^,^ ^26^ ^,^ ^40^ ^,^ ^41^ ^)^ corroborated our results, pointing to the need for adjustments on these tools when applied to different samples.

As to the structural model tested, appetite, symptoms of the disease, presence of metastasis, sex, and socio-economic class were important for quality of life. The impaired appetite and symptoms resulting from the disease had a highly significant influence on the patients’ quality of life. INCA^(^ ^27^ ^)^ provides specific nutritional recommendations for cancer patients with low appetite and/or presence of other symptoms. Therefore, we suggest that these recommendations be rigorously considered in the management of cancer patients. The relation between the presence of metastasis and a greater impairment in the quality of life can be attributed to the fact that this condition increases the patient’s weakness, and intensifies the intervention/treatment, resulting in greater or, in some cases, more aggressive side effects of the disease.

In our study, we observed that women with cancer and individuals with better financial conditions had worse quality of life. Lopes et al.,^(^ ^42^ ^)^ and Gijsberts et al.,^(^ ^43^ ^)^ suggested that, in the course of some diseases, the quality of life is more affected in women than in men. These authors attributed this fact to non-biological aspects, pointing out that women are psychologically more susceptible to environmental stressors than men, with a greater burden of physical and environmental stress, mainly due to the difficulty in maintaining their routine functions. Regarding socio-economic class, our results were opposite to existing literature, and any further discussion would be speculative, because a more in-depth investigation of the social and economic indicators of the sample should be conducted to explain this fact.

The analysis of the results should consider some limitations of the study, such as the lack of a more accurate dietary assessment, using different tools, and a more in-depth evaluation of the patients’ clinical status. Despite this, this study sought to use an enlarged sample, which included individuals with different diagnoses, treatments and clinical conditions in an attempt to minimize these biases.

## CONCLUSION

The dietary intake of the cancer patients evaluated did not reach the recommended levels of energy and macronutrient intake, but this fact did not directly interfere with the impairment in quality of life reported by them. Appetite/symptoms, sex, presence of metastasis, and socio-economic class had a significant impact on the patients’ quality of life and should be considered in protocols for clinical decision making.
